# Community pharmacists’ knowledge, prospective and practice towards health related illness at Hajj and Umrah: A cross sectional study

**DOI:** 10.1016/j.jsps.2023.101786

**Published:** 2023-09-14

**Authors:** Sultan Alghadeer, Salmeen D Babelghaith, Wajid Syed, Mohamed N. Al-Arifi

**Affiliations:** Department of Clinical Pharmacy, College of Pharmacy, King Saud University, Riyadh, Saudi Arabia

**Keywords:** Community pharmacist, Knowledge, Illness, Hajj, Vaccine, Pilgrims

## Abstract

**Objectives:**

To assess the community pharmacists' (CPs) knowledge, attitude, and perception of health-related illness among pilgrims, and to investigate the common diseases and the pattern of medications dispensed by CPs during Hajj and Umrah seasons.

**Method:**

A cross-sectional study was carried out in Riyadh, Saudi Arabia over two months in 2022, through electronic platform using prevalidated questionnaires adopted from the literature. The questionnaires were divided into 4 sections assessing the CP's knowledge, attitude, and perception about health-related illness, common dispensed agents, and required vaccination during Hajj and Umrah.

**Results:**

A total of 544 CPs, mostly between the age of 31–40 (69.9%), participated in this research. About 87.9% of the CPs received a pilgrim after performing their rituals coming to the pharmacy complaining of infection or health problem. In this study, 99.8%(n = 544), 99.6%(n = 543), and 92.7% (n = 505) of the CPs identified influenza, food poisoning, and diarrhea/gastroenteritis as the most common issues during the Hajj & Umrah season respectively. As results, anti-diarrheal agents (96.3%), painkillers (87.3%), inhalers (89.4%), and sunscreens (88.3%) were the most requested pharmaceutical agents. Additionally, 96.7%(n = 527) of the CPs agreed that vaccination is safe to be given to Hajj and Umrah pilgrims particularly for those aged ≥ 65 years, and 89.4%(n = 487) of them reported awareness of vaccines that are required by Saudi Ministry of Health. Both Influenza and meningococcal meningitis vaccines were identified by 99.8% of the CPs, but polio vaccine was identified by 33.9%.

**Conclusion:**

Community pharmacists provided pharmaceutical care services for Hajj and Umrah pilgrims. The majority of CPs had adequate knowledge about viral diseases during Hajj and Umrah and their requirement for vaccination.

## 1 Introduction

Millions of Muslims from all over the world visit Mecca and Madinah in Saudi Arabia each year during certain days of the last month of Islamic calendar to perform the Hajj (Aljohani et al., 2022, Dauda Goni et al., 2019). Umrah can be conducted at any time of the year, and this trip draws millions of worshipers to Mecca (Aljohani et al., 2022, Dauda Goni et al., 2019). Major issues in public health and infection control arise during the Hajj because of the crowded presence of population in certain days at specified place which create a mass gathering environment. Many of the older Hajj pilgrims have underlying medical issues that necessitate further health and pharmaceutical care (Mushi et al., 2021).

Respiratory tract infections such as seasonal influenza, meningococcal disease, lower respiratory infections, and tuberculosis as well as hepatitis A, B, and C (Gautret et al., 2016, Goni et al., 2020, Almutairi et al., 2018) are among the reported infections during Hajj. The most frequent medical issue affecting Hajj pilgrims is upper respiratory tract infections (URTIs) (Yezli et al., 2021, Barasheed et al., 2013, Bokhary et al., 2020). Haemophilus influenza and Staphylococcus aureus were the most frequently reported bacterial causes, while human rhinoviruses were the most the most prevalent viral respiratory infections identified from unwell Hajj pilgrims (Bokhary et al., 2020, Hoang et al., 2019, AlTawfq and Benkouiten, 2018). Influenza, as a common viral infection among pilgrims, is caused by different strains over years (Alzeer, 2009). Additionally, Hajj as mass gatherings environment encourages the spread of infectious organisms, particularly those responsible for food- and water-borne illnesses. Gastroenteritis and diarrhea as results were commonly reported among Hajj pilgrimages (Yezli et al., 2021, Barasheed et al., 2013, Gautret et al., 2015).

The mass gathering (Hajj) is at higher risk for both bacterial and viral infections, thus pharmaceutical care services should be optimal during Hajj at all levels, especially at the level of community pharmacy where CPs can be easily accessed by hajj pilgrims (Alomi et al., 2015, Wiyanna and Baladad, 2020). The CPs play an important role during the pilgrimage season including preventing infections, providing pharmaceutical care for pilgrims and dispelling myths about the uses of medications in general and antibiotics in particular to prevent the development of bacterial resistance. Also, they can deliver crucial immunization and educate about screening and vaccination.

On contrast, a lack of pharmacist knowledge can restrict good counseling and proper treatment. This is because most patients, particularly in underdeveloped nations, seek treatment for respiratory tract infections from pharmacists (Syed et al., 2023, Sales et al., 2021, Bashatah et al., 2023; Samreen et al., 2023) rather than from doctors, primarily because of socioeconomic concerns (Abdelaziz et al., 2017). Since no recent studies that have been published to address this issue regarding the preparation of CPs for the Hajj and Umrah seasons, the goal of the current study was to assess the community pharmacists' knowledge, attitude, and perception of health-related illness among pilgrims during the Hajj and Umrah in Saudi Arabia. In addition, this study aimed to investigate common diseases during Hajj and the pattern of medications dispensed by CPs.

## 2 Methods

### 2.1 Study design, population, and setting

A cross-sectional study was carried out over two months (from November 1^st^ to December 31^st^, 2022) using electronic google forms for the data collection. The data was collected using prevalidated questionnaires adopted from the literature. The CPs who (1) currently reside in capital city of Saudi Arabia, (2) able to read and understand the questionnaires, and (3) willing to complete the survey by providing informed consent were included, while individuals who did not match the inclusion criteria were excluded from the study. All the participants were informed that the collected data would be used only for research purposes and information would be kept confidential. Since all of the community pharmacies' regional headquarters are in Riyadh, the Saudi Arabian capital was chosen as the sample's location. Additionally, pilgrims traveling to holy locations frequently stop in the capital to see the area or to pass through. We chose the sample from the Riyadh region since many local pilgrims did Umrah by traveling from Riyadh to holy cities, and upon their return, they might have been infected. The data was collected by approaching the headquarter managers of CPs from whom the active mobile number and email were collected, and electronic forms were distributed.

### 2.2 Sample size

Similar to many previous studies the required sample size was calculated using an online calculator, (https://www.raosoft.com/samplesize.html). There are approximately 8419 CPs working in Saudi Arabia (AlRuthia Y et al., 2018). The sample size was calculated at 95% CI, and 5% of margin of error, and the calculated sample size was 368 respondents.

The questionnaires used for this study as adopted from the recently published similar study (Dauda Goni et al., 2019, Hoang et al., 2019, Wiyanna and Baladad, 2020, Abdelaziz et al., 2017). The questionnaires were divided into four sections. Section one covers the demographic details with a total of 8 items including, gender, age, years of experience in the pharmacy, educational level, country of qualification, a question about received or seen a pilgrim come to the pharmacy complaining of infection, the status of providing hajj or Umrah-related advice, and frequency of providing health advice to pilgrims. The second part of the study collected information about the knowledge of CPs about the most common health-related illness in holy cities that are assessed on multiple-choice answers. The third section deals with the knowledge about influenza infection during the Hajj and Umrah and consisted of 4 items assessed on a three-point scale (yes /no /I don't know). The fourth section asked CPs about the most commonly prescribed medications, pharmaceutical agents, first aid items and most commonly given vaccines before traveling to Saudi Arabia. The last section collected information about the knowledge of CPs towards vaccination with a total of 6 items. After the initial draft of the questionnaires, it was subjected to validation. The validation involves a review of the first draft of the tool for content checking through research experts in the associated field. Then, a pilot study was conducted with a randomly selected sample of 20 respondents to obtain their feedback on how to make the questionnaire more user-friendly. The Cronbach’s alpha was estimated for reliability tests, and a score of 0.80 suggested that the questionnaires were useful for the study.

### 2.3 Data analysis

The Statistical Package for the Social Sciences (SPSS) (version 26 for Windows (SPSS Inc., Chicago, IL, USA) was used to analyze the data. The demographic characteristics were summarized using descriptive statistics. To examine the difference in the variables, univariate analysis (chi-squared test/Fisher’s exact test) was utilized. All statistical tests were conducted using a 0.05 significance level.

## 3 Results

A total of 544 CPs participated in this research. Most of the participants were male (92.8%) who were between the age of 31–35 years old (35.2%; n = 192), and between 36 and 40 years old (34.7%; n = 189). With regards to years of experience, 37.2% (n = 203) of them were having 6–10 years while 26.8% (n = 146) were having > 15 years of experience. More than half of the CPs (59.6%) graduated from Egypt, while one-third 33.2% of them graduated from Saudi Arabia. Detailed information about the demographics is presented in Table 1.

**Table 1:** Demographics characters of the CPs **(n = 544)**.

**Characters**	**Frequency (n)**	**Percentage (%)**
Gender Male Female	506 38	. 92.8 7.0
Age (years) 21–25 26–30 31–35 36–40 > 41	23 92 192 189 48	4.2 16.9 35.2 34.7 8.8
Years of experience in community pharmacy 0–5 years 6–10 years 11–15 years > 15	97 203 98 146	17.8 37.2 18.0 26.8
Educational level Diploma (pharmacy technician) Bachelor BPharm PharmD (Doctor of Pharmacy) Postgraduate studies (master's degree or Ph.D.)	0 244 176 124	-- 44.8 32.3 22.8
Country of qualification Saudi Arabia Egypt Yemen Pakistan India	181 325 17 8 13	33.2 59.6 3.1 1.5 2.4
Do you provide Hajj and Umrah-related advice to patients? Yes No	476 68	87.3 12.5
Have You seen or received a pilgrim come to the pharmacy complaining of infection? Yes No	479 65	87.9 11.9
How much time or days do you spend on providing Umrah related health advice to pilgrims? I did not provide travel advise Monthly basis Weekly basis	27 107 410	5.0 19.6 75.3

In this study, 87.3% (n = 476) of the CPs agreed that they provide Hajj and Umrah-related health information to their customers. About 87.9% (n = 479) of the CPs agreed that they saw or received a pilgrim after performing their rituals coming to the pharmacy complaining of infection or health problem. Regarding the time spent on providing health-related advice by pharmacists to their customers, 75.3% (n = 410) of the CPs reported providing health-related advice on weekly basis, while 19.6 %(n = 107) on monthly basis and 5% of them did not provide any advice at all, respectively. The detailed information is given in Table 1.

**Fig. 1:**
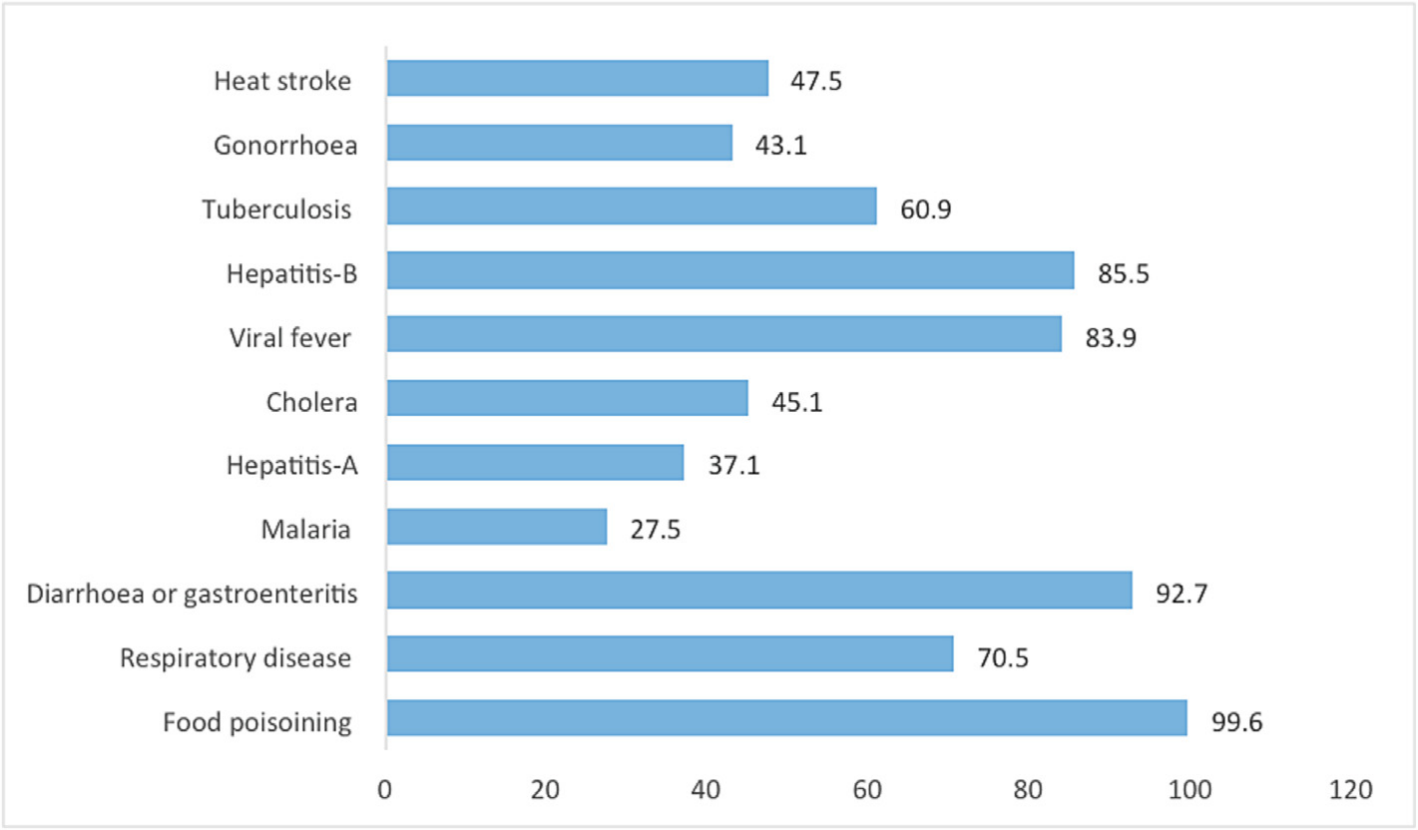
Knowledge of community pharmacists about most common health-related infection during the Hajj & Umrah.

In this study, 99.6%(n = 543) and 92.7% (n = 505) of the CPs identified food poisoning and diarrhea/gastroenteritis as the most common issues during the Hajj & Umrah season, Also, Hepatitis-B (85.5%; n = 466) and viral fever (83.9%; n = 456) were reported as common illness. Detailed information of the most common infections at holy cities is given in Fig. 1.

The majority 99.8%(n = 544) of participants agreed that Influenza can transmit easily from person to person during Haj and Umrah, and classified as the most common viral illness. However, the majority of the CPs 77.8%(n = 424) did not agree that younger adults have a higher risk of complications from influenza and other Hajj and Umrah health-related conditions. Additionally, 73.9% of the CPs did not agree that children, adults, and the elderly have the same risk of infection during the Hajj and Umrah seasons. Detailed knowledge of influenza infection during the Hajj and Umrah is given in Table 2.

**Table 2:** Knowledge about influenza infection during the Hajj and Umrah.

**Characters**	**Frequency (N)**	**Percentage (%)**
Influenza is common among hajj and Umrah travelers Yes No I don’t know	544 1 0	99.8 0.2 0
Younger adults have a higher risk of complications from influenza and other infections Yes No I don’t know	14 424 106	2.6 77.8 19.7
During Haj and Umrah, influenza can readily spread from person to person. Yes No I don’t know	544 1 0	99.8 0.2 0
During the Hajj and Umrah seasons, children, adults, and the elderly are all at danger of contracting influenza. Yes No I don’t know	101 403 40	18.5 73.9 7.3

**Fig. 2:**
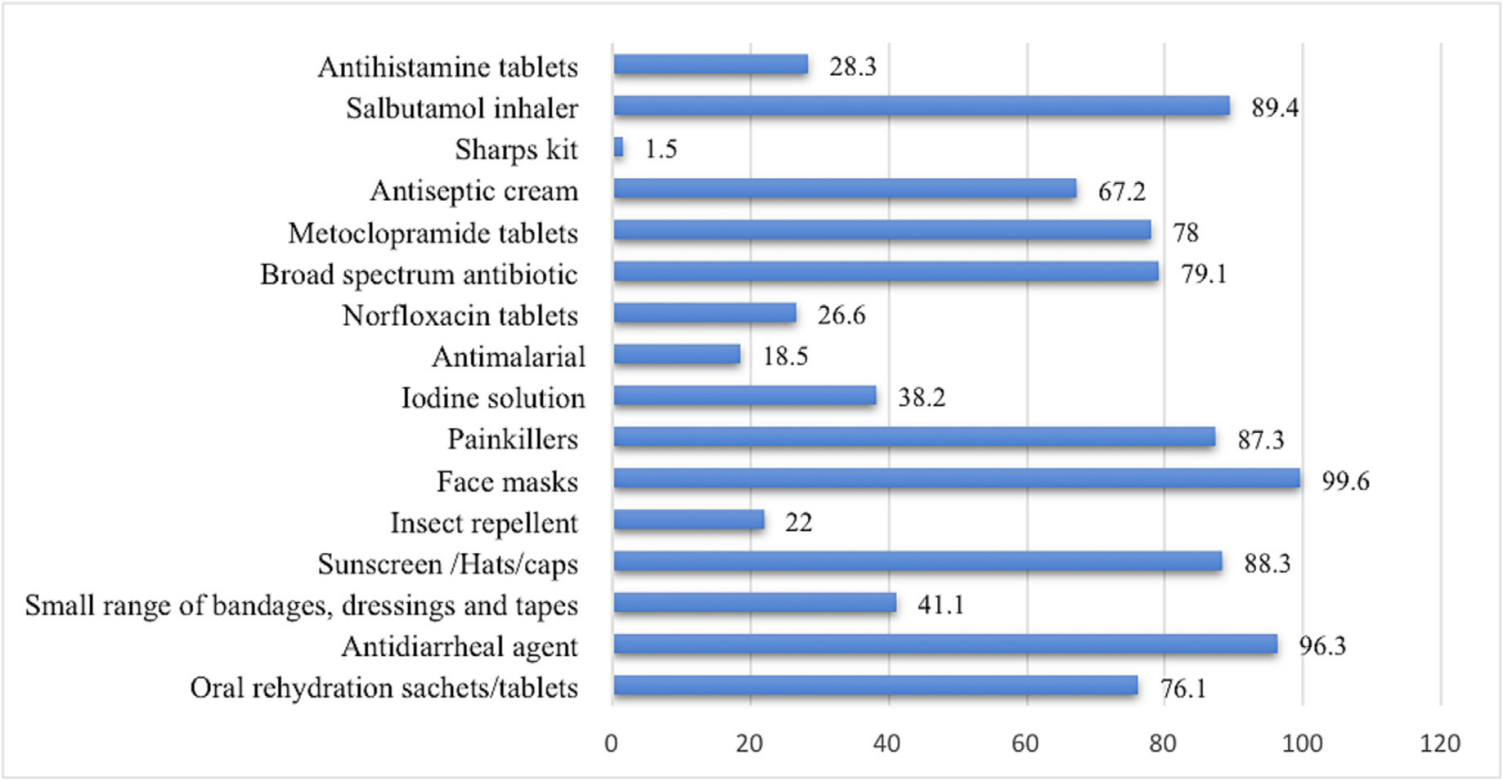
Most requested or recommended agents during Hajj and Umrah.

**Fig. 3:**
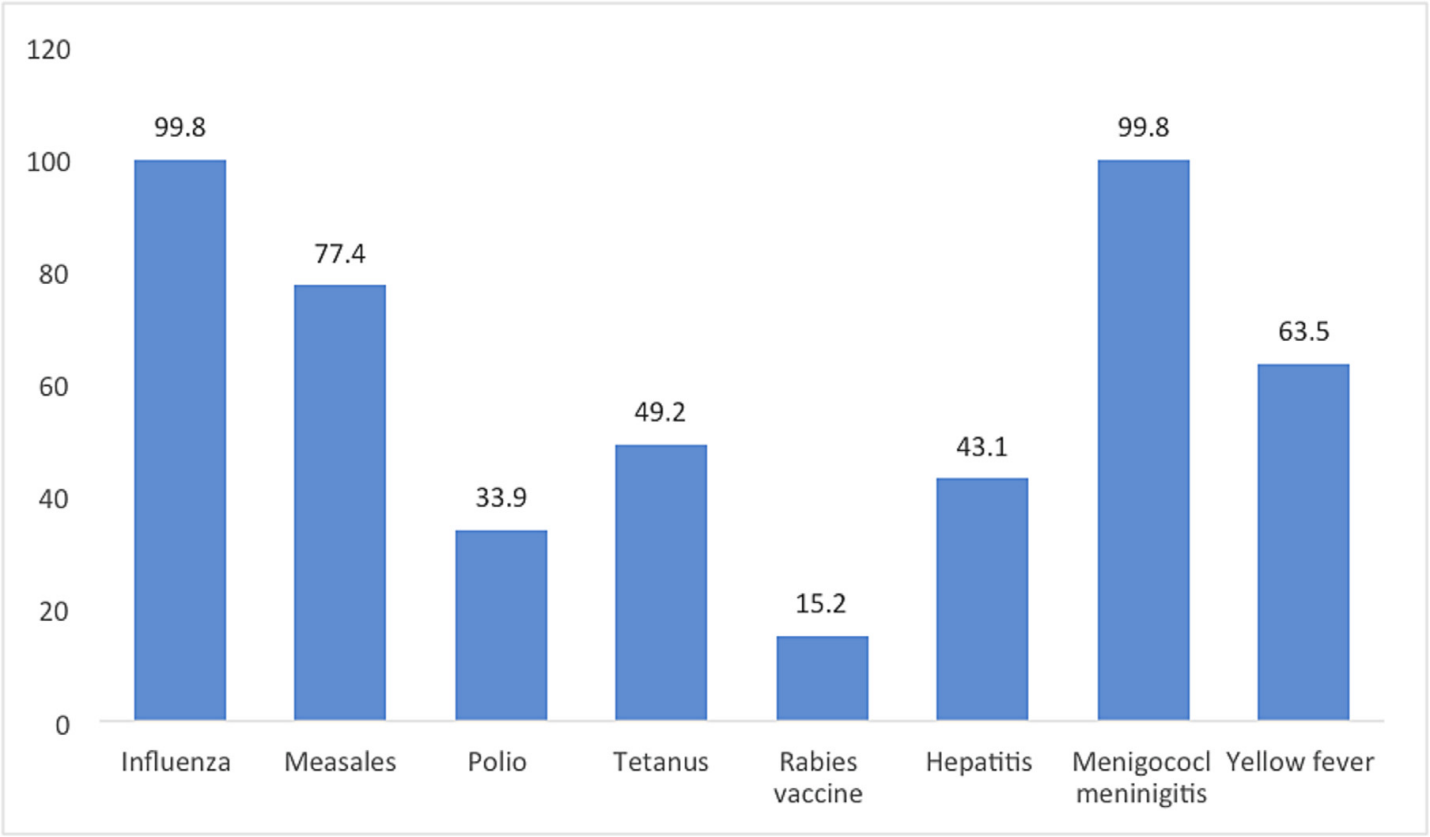
Knowledge of most familiar vaccines to be taken before traveling to Saudi Arabia.

With regards to recommending the items to their customers during the Hajj and Umrah seasons, most of the CPs recommended face-masks 99.6%(n = 543), followed by anti-diarrheal agents 96.3%(n = 525), over-the-counter painkillers 87.3%(n = 476), inhalers 89.4%(n = 487) and sunscreens 88.3%(n = 481). Detailed information about the CPs recommending the kits for the pilgrims is given in Fig. 2. With regards to the most familiar vaccines that must be taken before traveling to Saudi Arabia, CP mostly identified the followings: influenza vaccine 99.8%(n = 544), meningococcal meningitis vaccine 99.8%(n = 544), measles vaccine 77.4%(n = 422) yellow fever vaccine 63.5%(n = 346) (Fig. 3).

CPs’ knowledge regards to vaccines and their safety are presented in Table 3. In this study, 96.7%(n = 527) of the CPs agreed that vaccination is safe to be given to Hajj and Umrah pilgrims aged ≥ 65 years. Also, 97.4%(n = 531) of the CPs believed that vaccination can save medical costs during the Hajj and Umrah seasons. Additionally, 70.5% of the CPs reported that updating immunization against vaccine-preventable diseases in all travelers is strongly recommended for safe Haj and Umrah, while 57.1% of the CPs reported that influenza vaccination for Hajj and Umrah performers should be done twice a year. Knowledge regards awareness of vaccines that are required by Saudi Ministry of Health (MOH) for pilgrims, 89.4%(n = 487) of the CPs reported that they were aware of the required vaccines for Hajj and Umrah Health. Furthermore, information is given in Table 3.

**Table 3:** Knowledge of community pharmacists about the vaccination.

**Characters**	**Frequency (N)**	**Percentage (%)**
Vaccination is safe for Hajj and Umrah pilgrims, especially the elderly. Yes No I don’t know	527 1 17	96.7 0.2 3.1
Vaccination can save medical costs Yes No I don’t know	531 1 13	97.4 0.2 2.4
All tourists should be immunized against vaccine-preventable diseases in order to ensure their safety while performing haj and Umrah. Yes No I don’t know	384 1 160	70.5 0.2 29.4
For protection and to stop the spread of infectious diseases, the vaccine is advised. Yes No I don’t know	536 1 8	. 98.3 0.2 1.5
Do you aware of the required vaccines for Hajj and Umrah Health? Yes No I don’t know	487 1 57	89.4 0.2 10.5
Influenza vaccination for Hajj and Umrah performers should be done twice a year Yes No I don’t know	311 36 197	57.1 6.6 36.1

## 4 Discussion

Pharmacy professionals are vital contributors to the healthcare team. They can also have a significant impact on the community by making pharmaceutical items accessible and raising public awareness of health issues (Basheti et al., 2021). CPs have consistently been the most approachable medical professionals. A proof of this is the fact that they continue to provide direct patient care despite the government's restrictions put in place as a result of the outbreak (Yılmaz and Şencan, 2021). Reliable advices convey from CPs to their customers during the direct patient-pharmacist interaction (Alshahrani, 2020).

The present study found that the vast majority of CPs reported the gastrointestinal related illness (99.6% for food poisoning and 92.7% for diarrhea) as the most common illness. Despite of the presence of gastrointestinal related illness among pilgrims, its prevalence have been declined. It is reported to be 9.7% (n = 133/1363) in a cross sectional study that included pilgrims from 28 countries (Gautret et al., 2015). A systematic review for all gastrointestinal related illness during Hajj from 1980 till 2014 concluded that gastrointestinal disease as cause for health-facility admission decreased from 76.6% prior to 2002 to 12.4% beyond 2002 (Hoang and Gautret, 2018). Such changes are attributed to the advanced improvement in the infrastructure of holy cities and places that contributed in controlling temperature and optimizing sanitary of water and food supply. With the decrease of gastrointestinal related illness, the respiratory infection diseases become most common typical ailment among pilgrims and the main reason for inpatient stays and outpatient visits throughout the event (Bakhsh et al., 2015, Yezli et al., 2020).

In this study, 70.5% of CPs believed that respiratory tract infections are common among Hajj and Umrah pilgrims. In addition, almost all CPs (99.8%) reported that influenza is the most common among hajj and Umrah travelers. These outcomes are supported by previous studies, which showed that upper and lower respiratory diseases are prevalent among pilgrims, especially influenza is widespread among pilgrims (Alzeer, 2009, Alqahtani et al., 2015). A cross-sectional study was carried out by Yezli1 et al among pilgrims attending primary healthcare centers during the Hajj 2019. This study aimed to investigate the pattern of diseases presentations, and it found respiratory diseases (45%) constituted the most common frequently diagnosed disease among various diseases (Yezli et al., 2022). A recent study has reported that 93% of Hajj pilgrims experience respiratory symptoms, while 78% of examined samples from pilgrims were positive for at least one pathogen (Hoang et al., 2019). Furthermore, about 400,000 and 24,000 of which would suffer from upper respiratory symptoms and influenza respectively in one hajj season (Alzeer, 2009). Various influenza strains were identified throughout the years.

Currently, seasonal influenza vaccine is one of the required vaccinations that each pilgrim must take as guided by the Saudi MOH (Saudi MOH, 2022) and recommended by literature (Alfelali et al., 2019, Algarni et al., 2016). Almost all 99.8% of our participants believe and know influenza infection and vaccination is common and required. Additionally, Numerous studies have demonstrated that influenza vaccination protects Hajj pilgrims against infection, minimizes illness transmission to others, and lowers mortality and morbidity among pilgrims at risk (Alqahtani et al., 2015, Alfelali et al., 2019, Al-Tawfiq et al., 2016, Hamid et al., 2015). It helps to prevent about 70% of influenza cases in laboratory-based environment of healthy individuals, and it’s found to effective in about 43.4% (p = 0.01) of 1569 pilgrims from different nationalities (Alfelali et al., 2019). Pilgrims who are 65 years of age or older or who have chronic medical disorders are more likely to develop a serious case of influenza. The influenza vaccine showed to be beneficial in the high risk group, including elderly patients (>65 years old), since the incidence of disease were seen to be 14% in the vaccinated subjects versus 7% in the unvaccinated subjects (Alzeer, 2009).

This study found that most CPs claimed that updating immunization against vaccine-preventable diseases is highly recommended for all travelers for a safe Hajj and Umrah (70.5%) and is recommended for the protection and to prevent the spread of infectious diseases (98.3%). These results are consistent with the Saudi MOH recommendations. In addition to influenza vaccine, SARS-COV-2 (COVID-19) and meningococcal vaccination are required for all pilgrims while poliomyelitis and yellow fever vaccines are required for pilgrims from certain countries before traveling to Saudi Arabia for Hajj and Umrah (Saudi MOH, 2022). About 89.4% of our participants admitted the awareness of the required vaccinations for Hajj and Umrah. However regardless of vaccine requirement specifications, 99.8% and 63.5% of the CPs identified meningococcal meningitis and yellow fever vaccines as required vaccines respectively while only 33.9% identified the poliomyelitis vaccine. Due to the frequent changes to SARS-COV-2 (COVID-19) vaccine requirements and policy, it was excluded from the questionnaire. Due to the importance of vaccination and easy access to community pharmacies, CPs' knowledge of vaccinations was assessed in this study. It was found that almost of CPs (97%) believed that vaccinations are safe and saves medical costs for Hajj and Umrah pilgrims. These beliefs about the safety and cost-effectiveness of vaccinations come along with fact that vaccine is introduced as the most safe and cost-effectiveness health intervention on long-term impact (Rodrigues and Plotkin, 2020, Kim, 2011).

The most common dispensed pharmaceutical items by our participated CPs are antidiarrheal agents (96.3%), salbutamol inhalers (89.4%), analgesics (87.3%), antibiotics (79.1%) oral rehydration sachets (76.1%), and antihistamine tablets (28.3%). There is no previous study that included the CPs. However, it may be compared initially with a study conducted on health centers and hospitals to determine the pattern of medication prescribed to Hajj pilgrims. This is consistent with data from hospitals and primary health care centers during the pilgrimage (Yezli et al., 2020, Yezli et al., 2022, Khan et al., 2018, Alzahrani et al., 2012). For instance, the most commonly prescribed medications to patients at 13 primary health care centers (PHCCs) in Mina (a holy place in Mecca where pilgrims perform and spend most of their religious procedures and times at) during the Hajj in 2018 were analgesics and antipyretics (79.4%), followed by antimicrobial agent (53.9%), and anti-cough (37.1%) (Alzahrani et al., 2012). Another study reported that the most frequently prescribed drugs for hospital outpatients during the 2018 Hajj were analgesics (22.8%), anti-inflammatory drugs (22.9%), and systemic antibiotics (17%) (Yezli et al., 2020). A more recent study conducted in 2022, revealed that analgesics (25.1%), antibiotics (16.5%), anti-inflammatory and antirheumatic drugs (16.4%), and cough and cold products (11.9%) were the most frequently prescribed classes at Mina PHCCs (Yezli et al., 2022).

Based on our study and abovementioned studies, it is noticeable that analgesics, anti-inflammatory drugs, antibiotics, and cough/cold products are amongst the most dispensed/prescribed items for pilgrims. On contrast, antidiarrheal agents are only commonly dispensed by our participated CPs despite of the decline of gastrointestinal related illness. Additionally, studies revealed that the majority of URTIs are treated with antibacterial drugs, even though up to 95% of URTIs at the outset of symptoms, during Hajj, are known to be viral (Bokhary et al., 2020). Furthermore, the high usage of antibiotics was seen in 61.8% of Malay pilgrims in 2013, 53.8% of French pilgrims in 2012, and 45–48.3% of Indian pilgrims in 2016 (Hoang and Gautret, 2018). These results are consistent with ours since the CPs reported 87.9% of pilgrims come to the pharmacy complaining of infection. Regardless of the true bacterial infection, the CPs should be the worth trusty easy accessible healthcare provider since 66.5% of them spends between less to more than one hour weekly providing counseling on medication and health-related conditions to 87.3% Hajj pilgrims.

The methodology could be a limitation of our study. However, the busiest schedule and heavy daily tasks of CPs, and the nature of gathered information that is usually undocumented interaction and intervention between the pharmacist and client necessitate the choice of online cross sectional questionnaire method. Another limitation is the selection of CPs who works in Riyadh province. However according to the statistics of Hajj at 2019 (the year before COVID-19 pandemic), there were 2,489,406 pilgrims; 33% of them were domestic pilgrims and most of which came from outside of Mecca (GASTAT, 2019). Another possible limitation is that this study assessed time and counseling advice by CPs for pilgrims, but it didn’t assess the satisfaction of such services by pilgrims. Lastly, nature of cross-sectional study may have recall bias.

## 5 Conclusion

The current study revealed that the majority of CPs have good knowledge about most common disseminated diseases and requested pharmaceutical agents during the Hajj and Umrah, and they are familiar with the vaccination requirements.

## Declaration of Competing Interest

The authors declare that they have no known competing financial interests or personal relationships that could have appeared to influence the work reported in this paper.
